# Emergence of colistin-resistant hypervirulent *Klebsiella pneumoniae* (CoR-HvKp) in China

**DOI:** 10.1080/22221751.2022.2036078

**Published:** 2022-03-03

**Authors:** Xiaoyu Liu, Yarong Wu, Ying Zhu, Peiyao Jia, Xue Li, Xinmiao Jia, Wei Yu, Yujun Cui, Ruifu Yang, Wei Xia, Yingchun Xu, Qiwen Yang

**Affiliations:** a Medical Technology Academy, Beihua University, Jilin, Jilin Province, China; Department of Clinical Laboratory, Peking Union Medical College Hospital, Peking Union Medical College, Beijing, People's Republic of China; b Department of Clinical Laboratory, State Key Laboratory of Complex Severe and Rare Diseases, Peking Union Medical College Hospital, Chinese Academy of Medical Sciences and Peking Union Medical College, Beijing, People's Republic of China; c State Key Laboratory of Pathogen and Biosecurity, Beijing Institute of Microbiology and Epidemiology, Beijing, 100071, People's Republic of China; d Graduate School, Peking Union Medical College, Chinese Academy of Medical Sciences, Beijing, People's Republic of China; e Department of Clinical Laboratory, Beijing Anzhen Hospital, Capital Medical University, Beijing, China; f Medical Research Center, State Key Laboratory of Complex Severe and Rare Diseases, Peking Union Medical College Hospital, Chinese Academy of Medical Sciences and Peking Union Medical College, Beijing, People's Republic of China; g Central Research Laboratory, Peking Union Medical College Hospital, Peking Union Medical College, Chinese Academy of Medical Sciences, Beijing, People’s Republic of China

**Keywords:** *Klebsiella pneumoniae*, *mgrB*, colistin-resistance, hypervirulence, ST11

## Abstract

Colistin is regarded as a last-resort agent to combat infections caused by multidrug-resistant (MDR) Gram-negative bacteria, especially carbapenem-resistant isolates. In recent years, reports of colistin-resistant *Klebsiella pneumoniae* (CoRKp) are increasing. However, the molecular mechanism and relevance of colistin resistance and virulence remain unclear. Fourteen CoRKp strains were retrospectively screened from 1884 clinical *K. pneumoniae* isolates during 2017–2018 in China. Six CoRKp strains belonging to ST11 were MDR strains. Plasmid-mediated mobile colistin-resistance genes had a low prevalence in CoRKp. Our results revealed that up-regulated expression of two-component systems, especially *phoPQ,* contributed more to colistin resistance. *mgrB* mutation was the most common molecular mechanism of colistin resistance, caused by either nonsense mutations or insertion sequences, which drove the overexpression of *phoPQ system*. This study also identified three novel point mutations in *pmrAB* system, in which D313N mutation in *pmrB* was proved to increase the MIC to colistin by 16-fold. In addition, 6 out of 14 CoRKP strains independently carried hypervirulence genes. All six strains showed medium-to-high virulence phenotype compared with NTUH-K2044 strain in mice intraperitoneal challenge models. We found that 4 strains were biofilm strong producers and transcriptome analysis revealed that three of them significantly up-regulated expression of type III fimbrial shaft gene *mrkA*. In conclusion, our result revealed the emergence of colistin-resistant and hypervirulent MDR *K. pneumoniae,* which is a noticeable superbug and could cause a severe challenge to public health.

## Introduction

Colistin was a human medication to treat clinical infections before the 1950s, while it was limited to be used only in agriculture and graziery due to its renal toxicity and neurotoxicity [1]. Recently, with the emergence of multidrug-resistant (MDR) gram-negative bacteria, particularly carbapenem-resistant Enterobacteriaceae (CRE) all over the world, colistin returned to the clinical field as “the last option” to treat carbapenem-resistant *Klebsiella pneumoniae* (CRKP) and other MDR pathogenic bacterial infections [2]. At the same time, reports of colistin-resistant *Klebsiella pneumoniae* (CoRKp) have been increasing, raising a significant challenge to healthcare [1,3,4].

In *K. pneumoniae,* lipopolysaccharide (LPS) modification leads to the reduction of LPS negative charge, which reduces its affinity to colistin, resulting in colistin resistance [5]. Plasmid-mediated mobile-resistance genes (*mcr-1* to *mcr-10*) and chromosome-mediated regulation pathways are two main mechanisms of LPS modification [5,6]. The MCR protein, a phosphoethanolamine (PEtN) transferase enzyme encoded by *mcr* gene, could catalyze the addition of PEtN to lipid A and result in a more cationic LPS [7]. Meanwhile, mutations of genes in *pmrAB* and *phoPQ* two-component systems (TCS), or their regulators (such as *mgrB*) could increase the addition of 4-amino-4-deoxy-l-arabinose (l-Ara4N) and PEtN to lipid A in LPS, and consequently elevate the MICs [8].

Hypervirulent *K. pneumoniae* (hvKp) was usually reported as a life-threatening pathogen. The sequence and serotype of hvKp were often reported as ST23-K1 and ST86/65-K2. Compared with the classic *K pneumoniae*, hvKp was usually less relevant with resistance [9]. As the most prevalent MDR lineage in Asia [10], *K. pneumoniae* of ST11 was first reported to become a hypervirulent strain in China [11]. There have been increasing reports of MDR *K. pneumoniae* with virulence genes and related fatal hospital outbreaks [11–13], indicating the harm of MDR hvKp.

In this study, we retrospectively identified 14 CoRKp strains from 1884 non-repetitive clinical *K. pneumoniae* isolates in China during 2017–2018 Study for Monitoring Antimicrobial Resistance Trends (SMART) surveillance. Molecular and genetic analysis revealed that chromosome-mediated resistance was the predominant mechanism of CoRKp. Thus, we focused on investigating the impacts of transcription and genetic variants of genes involved in TCSs and their regulators mentioned above on colistin resistance. In addition, our results revealed the cooccurrence of colistin resistance and hypervirulence in *K. pneumoniae*, especially in ST11 lineage. These findings provide evidence that high-level colistin-resistant MDR *K. pneumoniae* could become hypervirulent and hint at the importance of monitoring colistin-resistance mechanism and virulence factors of CoRKp.

## Materials and methods

### Stains and antimicrobial susceptibility testing

From 2017 to 2018, 1884 non-repetitive *K. pneumoniae* strains from patients of 22 multi-centre tertiary class-A hospitals in China were collected in the SMART surveillance project [14]. All isolates were sent to Peking Union Medical College Hospital for re-identification by using Vitek MALDI-TOF MS (BioMérieux, France). The minimum inhibitory concentrations (MICs) of polymyxin B and colistin were determined by broth microdilution (BMD) which is the gold-standard reference method according to the Clinical and Laboratory Standards Institute (CLSI) M100-ED30 [15]. Considering that the standard has replaced the resistance breakpoints with intermediate, we used the Antimicrobial Susceptibility Testing Breakpoint v10.0 [16] made by the European Committee to facilitate calculation. The polymyxin B and colistin MICs for *K. pneumoniae* were interpreted in this manner: susceptible ≤ 2 mg/L, resistant > 2 mg/L, with *Escherichia coli* ATCC 25922, *Pseudomonas aeruginosa* ATCC 27853 and colistin-resistant *E. coli* NCTC 13846 (*mcr-1* positive) as quality control strains. Antimicrobial susceptibility testing for other drugs was performed by BD Phoenix^TM^ M50 (Becton Dickson Diagnostics, USA).

### Whole-genome sequencing and assembly of colistin-resistant isolates

We used TIANamp Bacteria DNA Kit (DP302) to extract genomic DNA from colistin-resistant isolates, which were inoculated in LB medium (Sangon Biotech, China), and cultured at 37°C for 18 h. Then we quantified and qualified the yield of extracted DNA by NanoDrop 2000 and Qubit 2.0 Fluorometer. Whole-genome sequencing was performed on Illumina Novaseq6000 (Illumina Inc, USA) with 150-bp paired-end library. The raw sequencing reads were trimmed by Trimmomatic v0.38 [17] and then assembled *de novo* using Shovill v1.0.1 (https://github.com/tseemann/shovill) with a minimum contig length of 200.

### Multi-locus sequence typing (MLST) and O and K antiserum typing (serotypes)

The assembled genomes were used for in silico multi-locus sequence typing (MLST) and O and K antiserum typing. MLST analysis was conducted using MLST software (https://github.com/tseemann/mlst) with contig files compared against traditional PubMLST typing schemes [18]. Meanwhile, O and K serotypes were determined by Serotype Finder v2.0.1 (https://cge.cbs.dtu.dk/services/SerotypeFinder/), based on sequence alignment against its public database v1.0.0 [19].

### SNP calling and phylogeny reconstruction

A total of 46 genomes were used in the phylogeny reconstruction, including 14 newly sequenced genomes of colistin-resistant isolates, and 32 public sequences downloaded from NCBI, of which 31 genomes were selected from major clones of *K. pneumoniae* population, referred in Wyres *et al.* [20]. The public genome of NTUH-K2044 (Accession: NC_012731.1) was used as a reference.

All genomes were aligned against the reference genome using MUMmer (v3.0) [21] to generate whole-genome alignments and to identify single-nucleotide polymorphisms (SNPs) in the core genome, with repetitive regions removed. In total, 126,058 SNPs were identified in these 46 genomes. Based on the concatenated SNPs, a maximum-likelihood tree with 1000 fast bootstrap replicates was inferred using IQ-TREE under GTR + G4 substitution model [22].

### Identification of antibiotic-resistance genes

ResFinder v4.0 [24]; and PointFinder [23] were used to identify antibiotic-resistance genes or mutations with a minimum coverage of 80% and a minimum identity of 80%. As reported, variations located in coding regions and upstream intergenic regions of the TCS and transferase genes including *pmrABCK, phoPQ*, *crrAB*, and its negative regulator *mgrB* could be related with chromosome-mediated colistin resistance through its effects on transcription*.* We recalled SNPs for these genes and identified insertion sequences involved in *mgrB* gene based on BLAST alignment against the reference genome of *K. pneumoniae* NTUH-K2044. *K. pneumoniae* MGH 78578 (Accession no. NC_009648.1) was also used as a reference, due to that *crrAB* genes were accessory genes and absent in NTUH-K2044. Nonsynonymous and nonsense substitutions and insertion mutations were selected for further analysis. Meanwhile, plasmid-mediated mobile colistin-resistance genes (*mcr-1* to *mcr-10*) were screened by both multiplex PCR and genome comparison [24–27]. Specific *mcr* typing was finally determined by aligning assemblies against different *mcr-1* to *mcr-10* gene sequences in the public database of NCBI AMRFinderPlus [28] using blastn v2.2.28+.

### Chromosomal regulon gene expression analysis using real-time quantitative PCR (RT-qPCR)

The expression of chromosome-mediated colistin-resistant genes was detected to explore the relationship between regulon and resistance. A total of 41 *K. pneumoniae* isolates were divided into the colistin-resistant group (COL-R) and colistin-susceptible group (COL-S) based on their colistin MICs. Briefly, all isolates were grown to the logarithmic phase, and then their total RNA was extracted using RNA pure Bacteria Kit (DNase I) (CoWin Biosciences, China). Reverse transcription was performed using FastKing RT Kit (With gDNase) (TIAGEN, China) to produce cDNA from 1500 ng of total RNA for each isolate. Finally, the gene expression data were obtained by RT-qPCR using LightCycler 480 II (Roche, Switzerland) and TB Green Premix DimerEraser^TM^ (TaKaRa, Japan). The housekeeping gene *rpoB* was used as the internal reference. Each experiment was repeated three times, and the relative expression of each gene in different strains was calculated, denoted by 2^−ΔΔCt^.

### Functional confirmation of pmrA and pmrB novel point mutation

The construction of point mutation was performed in *K. pneumoniae* SZS128 strain (the MIC of colistin was 0.5 mg/L and harboured wide-type TCS genes) using CRISPR-Cas9-mediated genome-editing method containing pCasKP-apr and pSGKP-spe plasmid [29]. Mutations of G611 T in *pmrA* gene, A853 T and G937A in *pmrB* gene were constructed through two-step genome-editing procedure. Firstly, deleting ∼400 bp sequences around the point mutation by co-transforming a linear donor sequence and the spacer-introduced pSGKP-spe plasmid, individually, into the l-arabinose-induced pCasKP-apr-harbouring cells to partially delete the target sequence. After gene partial deletion, the pSGKp plasmid was cured by culturing in the LB agar plate containing 50 mg/L apramycin and 5% sucrose at 30°C. Secondly, the sequence containing point mutation was complemented into the cells using pSGKP whose spacer was located in the interfaces of upstream and downstream sequences of deleted sequences in the first step. The MICs of colistin were evaluated in the colony in which successful point mutation was confirmed by both PCR and sequencing after pCasKP and pSGKP plasmid curing. Preparation of competent cells, electroporation and spacer cloning into pSGKP plasmid were performed as Wang et al. [29] described. The primers used in this study were listed in Sup Table 2. BMD was used to detect the MIC of colistin against transformants.

### Identification of virulence genes

The virulence gene profiles of the 14 colistin-resistant strains were characterized through sequence alignment against the VFDB database with a minimum coverage of 80% and a minimum identity of 80% [30]. Meanwhile, the 14 genomes were also aligned against pLVPK plasmid genome (Accession: NC_005249.1) using blastn v2.2.28 + to detect the presence and absence of hvKp-related genes, including *rmpA*, *rmpA2*, *iucABCD*, *iutA*, *iroBCDN,* and *peg-344*, and to determine whether the isolates were hvKp or not. Finally, BRIG v0.95 software was used to visually display the plasmid genome comparison results, including six identified hvKp isolates, along with the plasmid genome of NTUH-K2044, and the reference pLVPK.

### Mouse intraperitoneal infection models

The animal protocols were reviewed and approved by the Animal Ethics Committee and Administration Institutional Animal Care Committee of Tsinghua University. All experiments were carried out in Tsinghua University Animal Biosafety Level 2 (ABSL-2) under the guidelines of the “Ethics of Animal Experimentation Statement.”

ICR female mice at the age of six orseven weeks were obtained from Vitalriver. Bacteria were grown in LB broth until logarithmic phase and stored in −80°C. Mice intraperitoneal (IP) infection models were modified from the previously published studies [31,32] to identify the MDR hypervirulence *K. pneumoniae*. NTUH-K2044 was used as a hypervirulent control strain, and QD110, a strain confirmed by whole-genome sequencing that did not contain any virulence genes, as a low-virulence control strain. Five mice were injected with 5×10^7^ CFU in a group of NTUH-K2044 and other experimental strains, or with approximately 1×10^8^ CFU of QD110, respectively. Then the mortality of mice was observed up to seven days.

### Biofilm formation

In order to reveal biofilm formation in CoRKp, we used crystal violet to analyze the biofilm-production capacity. Biofilm formation protocol was modified from the previous protocol [33] by using 1% crystal violet to dye the biofilm, and 96% ethanol to solubilize the dyestuff and then determined optical density at 590 nm. Three biological replicates were performed to ensure that all results were valid.

Biofilm formation capabilities were evaluated according to a previous report [34]. Three standard deviations above the mean OD of the negative control were regarded as the OD cut-off (ODc). Biofilm formation capabilities were classified into the following categories: non-biofilm capabilities (OD ≤ ODc), weak biofilm capabilities (ODc < OD ≤ 2ODc), moderate biofilm capabilities (2ODc < OD ≤ 4ODc), and strong biofilm capabilities (4ODc < OD).

### Transcriptome and data analysis

Total RNA was extracted using RNA pure Bacteria Kit (DNase I) (CoWin Biosciences, China) from three biofilm-positive strains, and one colistin-susceptible and weak biofilm strain, ATCC13883, which was used as control. Purification was performed under the manufacturer’s instructions. Each strain had three replicates. RNA-seq was performed on Illumina Novaseq6000 (Illumina Inc, USA) with 150-bp paired-end library. After trimming raw reads using Trimmomatic software, we aligned the reads to the reference genome of NTUH-K2044. Then, HTSeq (v0.6.1) [35] was used to annotate and count aligned reads for each gene. Differentially expressed genes (DEGs) were identified using DESeq2 [36] with thresholds of absolute log_2_^fold change^ >1 and Benjamini–Hochberg adjusted *P*-value (p. adjust) < 0.05. DEGs were displayed in volcano plot using the R package ggrepel. Finally, we used clusterProfiler [37] to analyze gene clusters for up-regulated and down-regulated DEGs in Kyoto Encyclopedia of Genes and Genomes (KEGG) pathways.

### Statistical analysis

Linear correlation between transcription level of chromosomal regulators and colistin MICs was analyzed in GraphPad Prism 8. Mann–Whitney *U*-test Spearman’s correlation and multiple linear regression were performed by SPSS v25.0. Mantel–Haenszel *χ*^2^-test was used to test association between virulence and MICs. Log-rank test (Mantel–Cox) was used to analyze the survival curve of mice infection models in GraphPad Prism 8. *P*-value <0.05 was regarded to be statistically significant, and *P* < 0.001 was extremely significant.

## Results

### Prevalence of colistin-resistant K. pneumoniae during 2017–2018 surveillance

A total of 1884 non-repetitive clinical *K. pneumoniae* were collected during 2017–2018 SMART surveillance survey with 22 hospitals from seven regions (north, 8.7%; northeast:16%; central, 8.2%; east non-Jiangzhe, 20.7%; east Jiangzhe, 20.1%; south: 9.4%; southwest, 16.9%) in China. The proportion of clinical isolates from intra-abdominal tract, respiratory tract, urinary tract, and blood were 24.2%, 45.0%, 17.1%, and 13.7%, respectively. Medical intensive care units (ICUs) collected 20.6% isolates, including general unspecified ICU (7.5%), surgery ICU (6.1%), medicine ICU (5.1%), and pediatric ICU (2.0%). The age of patients ranged from 0 to 101 years, > 60 years accounting for 54.8%, 31–60 years 38.5%, and < 30 years 6.8%.

Antimicrobial susceptibility testing was reperformed for 14 CoRKp strains. The prevalence of CoRKp was 0.74% (*n* = 14/1884). These strains were collected from seven provinces, five of them isolated from Zhejiang Province (see Table 1). The colistin MICs range of CoRKp was 4 to > 64 mg/L with a wide variety of specimen types including sputum (28.6%), bile (21.4%), blood (14.3%), and other sterile body fluids (see Table 1).

**Table 1.** Antimicrobial susceptibility profiles, isolates information and biofilm-production ability of colistin-resistance *K. pneumoniae*.

Name	Province	Gender	Age	Specimen type	MLST	Serotype	Hypervirluence factors	COL	AMK	FEP	FOX	CAZ	CRO	CIP	ETP	TGC	SXT	Biofilm formation (OD590nm)
A155	Hunan	M	59	bile	ST5253	K28	*iroB iucA*	4	≤8	≤1	≤4	≤1	≤1	≤0.5	≤0.25	≤1	>4/76	0.190657
A212	Shandong	M	69	Abdominal fluid	ST111	K63		4	≤8	16	≤4	2	>32	2	≤0.25	4	>4/76	0.412057
A141	Shandong	M	64	Sputum	ST15	K112		8	≤8	8	≤4	16	>32	>4	≤0.25	4	≤1/19	2.899057
A144	Hubei	F	1	Urine	ST2459	K15		16	≤8	≤1	≤4	≤1	≤1	1	≤0.25	2	>4/76	0.611657
A231	Shanghai	M	62	Pleural fluid	ST11	K64		16	>32	>16	>16	>32	>32	>4	>128	2	≤1/19	1.499457
A128	Jilin	F	55	Urine	ST15	K102		32	>32	>16	8	>32	>32	>4	≤0.25	2	>4/76	0.8732
A214	Hubei	M	58	bile	ST395	K48		32	>32	8	>16	>32	>32	>4	0.5	4	>4/76	1.2178
A218	Zhejiang	M	56	bile	ST3812	K107		32	≤8	>16	16	16	>32	2	4	≤1	≤1/19	0.493
A221	Zhejiang	F	66	Sputum	ST11	K64	*iroB iucA rmpA rmpA2*	64	≤8	>16	>16	32	>32	>4	>128	4	>4/76	0.7214
A224	Zhejiang	M	69	Blood	ST11	K64	*iroB iucA*	64	>32	>16	>16	>32	>32	>4	128	8	>4/76	0.3552
A225	Zhejiang	F	66	Sputum	ST11	K64	*iroB iucA rmpA rmpA2*	64	≤8	>16	>16	>32	>32	>4	>128	4	>4/76	0.7232
A140	Shandong	M	79	Sputum	ST86	K2	*iroB iucA rmpA rmpA2 peg344*	>64	≤8	≤1	≤4	≤1	≤1	≤0.5	≤0.25	2	≤1/19	0.0704
A30	Beijing	F	55	Urine	ST11	K47	*iucA*	>64	>32	>16	>16	>32	>32	>4	>128	2	>4/76	0.4552
A229	Zhejiang	F	47	Blood	ST11	K64		>64	≤8	>16	>16	>32	>32	>4	>128	2	>4/76	0.2916

Abbreviations: COL, colistin; AMK, Amikacin; FEP, Cefepime; FOX, Cefoxitin; CAZ, Ceftazidime; CRO, Ceftriaxone; CIP, Ciprofloxacin; ETP, Ertapenem; TGC, Tigecycline; SXT, Trimethoprim-Sulfamethoxazole

Antimicrobial susceptibility testing results also revealed that 64% (9/14) of the strains were resistant to cephalosporins (see Table 1), and 50% (7/14) resistant to carbapenem, carrying *bla*_KPC-2_ gene. Additionally, the tigecycline-resistance gene *tmexCD1-toprJ1* was identified in a tigecycline-intermediate isolate (see Sup. Figure 1 and Table 1).

Notably, 13 of 14 strains were MDR and four of them were extensively drug-resistant (XDR) isolates. One ST11 strain, isolated from the blood of a 69-year-old male in Zhejiang Province, was pan-drug-resistant (PDR), except for an intermediate resistance to chloramphenicol (see Table 1).

### Phylogenetic analysis of CoRKp and prevalence of mcr genes

**Figure 1.**
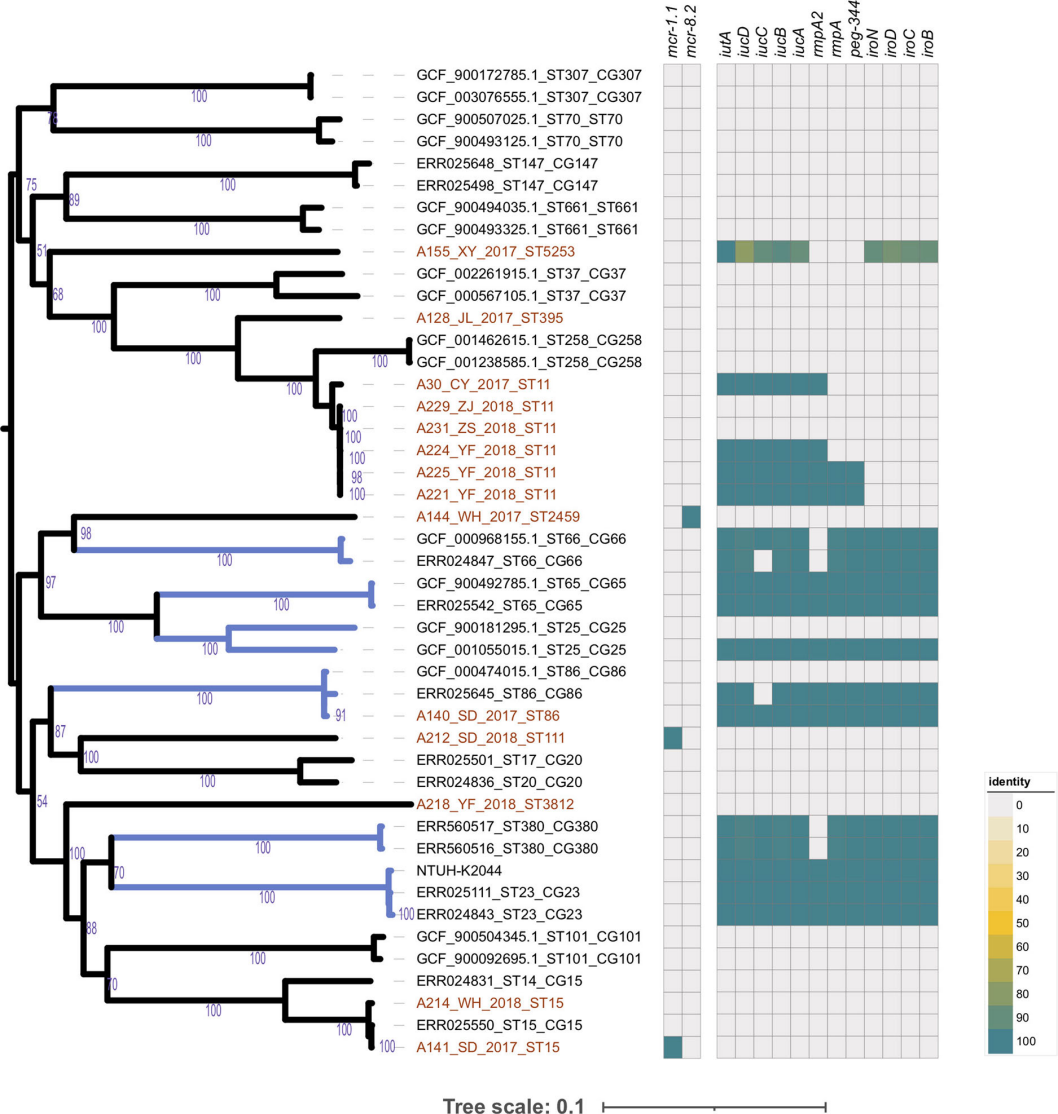
*K. pneumoniae* population structure and the prevalence of *mcr* gene and hvKp marker genes. The phylogeny in the left represents the population structure of 14 colistin-resistant isolated and 31 representative genomes of major global problem clones, with six hypervirulent clones (CG23, CG25, CG65, CG66, CG86, CG380) highlighted in blue branches, and 14 colistin-resistant isolated coloured in red text. Bootstra*p* values are also labelled on the nodes of the tree. The prevalence of *mcr* gene and hvKp marker genes are shown in the heatmap with whitespace separated. Different colors of the square in the heatmap represent the alignment identity for each gene (see the legend in the lower right section), with grey colour represents gene absence.

The phylogeny of newly sequenced colistin-resistant isolates was generated, together with representative strains of global problem clones of *K. pneumoniae* referred in Wyres *et al*. [20]. Phylogenetic analysis indicated that the 14 CoRKp strains had an extensive genetic diversity as they were assigned to eight lineages (see Figure 1). ST11 was the dominant sequence type of CoRKp (see Figure 1), with six genomes involved, of which four strains were isolated from Zhejiang Province. In addition, only one hypervirulent clone strain was CG86, and the rest were classified as ST15, ST111, ST395, ST2459, ST3812, and ST5253 scattering across the phylogenetic tree.

Only three isolates carried mobile colistin-resistance genes, which indicated that 21% (*n* = 3/14) of CoRKp isolates were *mcr-*positive. Sequence alignment showed that two isolates, A212 (ST111) and A141 (ST15), sampled from the same hospital of Shandong Province, harbouring *mcr-1.1* gene. A144 (ST2459) from Hubei Province, contained *mcr-8.2* gene (see Table 1 and Table 3). All three *mcr*-carrying isolates were colistin-resistant, with MICs of 4, 8, and 16 mg/L, respectively (see Table 3). The promoter sequences of *mcr-1.1* and *mcr-8.2* genes were also compared in these three sains, which could affect the expression of *mcr* gene and eventually result in various colistin MICs. Our results revealed variations between *mcr-1.1* and *mcr-8.2* promoters. However, the two *mcr-1.1* carrying strains with different MICs had identical genetic contexts (see Sup. Figure 4).

### Molecular mechanism of chromosome-mediated colistin resistance

**Figure 2.**
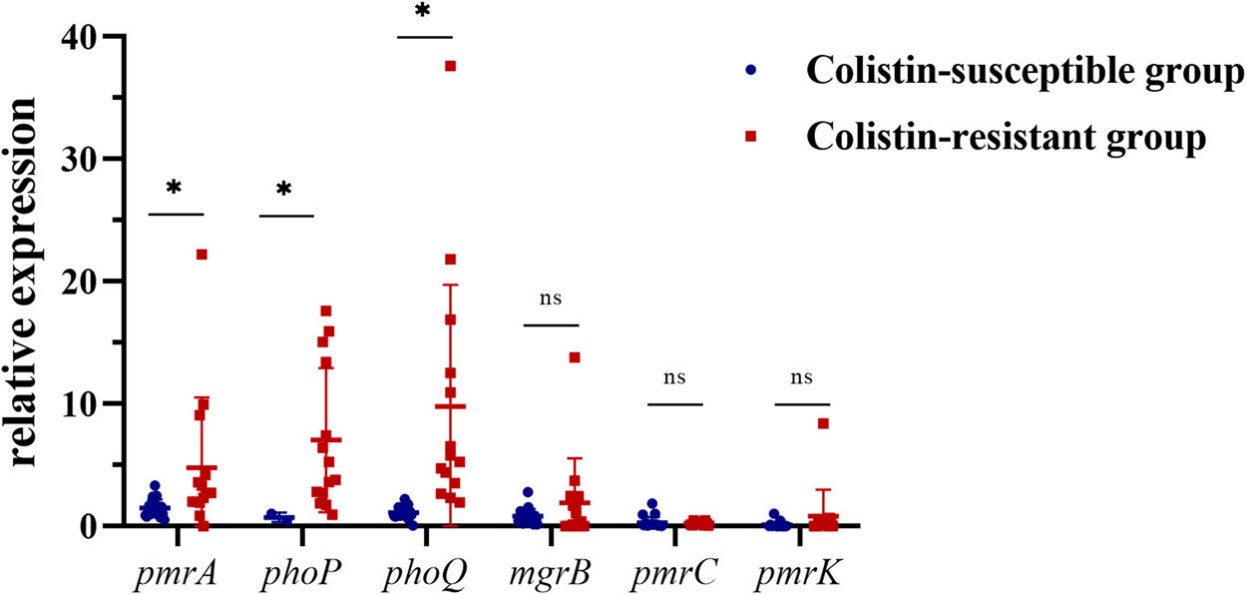
Relative expression of the *pmrA*, *phoP*, *phoQ*, *mgrB*, *pmrC,* and *pmrK* genes in colistin-resistant group and colistin-susceptible group. Values were represented by means of three independent replicates. Relative expression of *pmrA*, *phoP,* and *phoQ* in colistin-resistant group was significantly higher than colistin-susceptible group. There were 6 strains that less expressed *mgrB* gene, five strains harboured modified-*mgrB* gene and the rest strain were WT. However, because of early termination, overexpression of *mgrB* in one strain from ST11 could not form a protein with a negative regulatory function. *, *P* < 0.05; ns, not significantly different.

As only 3 CoRKp strains harboured plasmid-mediated colistin-resistance genes, we further studied the relationship among mutation, transcription, and colistin resistance based on *pmrA*, *pmrC*, *pmrK*, *phoP*, *phoQ,* and *mgrB*. Significant differences were found between resistant and susceptible groups in *pmrA*, *phoQ,* and *phoP* (Mann–Whitney *U*-test, *P* <0.05) (see Figure 2). The expressions of *pmrA*, *phoP,* and *phoQ* in all colistin-resistant strains were 3.23-fold, 9.64-fold, and 8.82-fold higher than colistin-susceptible strains. The results were also supported by Spearman’ correlation test, as there was high correlation coefficient between MICs and the expression of *phoP* (0.593, *P *< 0.05) and *phoQ* (0.766, *P *< 0.0001) (see Table 2). We used multiple linear regression to analyze the relationship between the transcriptional level of TCS and MICs, and obtained a formula, MIC = 4.121× *pmrA* + 3.501× *phoQ*-0.34 (*P* < 0.001). The influence coefficient of TCS on MICs was 0.691 (*P* < 0.0001). Moreover, we found that the transcriptional level of *phoQ* was positively correlated with *pmrA* (*R*^2 ^= 0.250) and *phoP* (*R*^2 ^= 0.541). Although there was no significant linear trend between *mgrB* and *phoP*, it still exhibited a negative correlation (see Sup Figure 5B). ST11 strains had no expression of *mgrB* gene except A231 expressed a low level. However, their expressions of *phoQ* were all elevated, which reflected the negative correlation between *mgrB* and *phoQ* in turn.

**Table 2.** Correlation coefficient between MIC and Chromosomal regulon gene expression.

			pmrA	phoQ	phoP	mgrB	pmrC	pmrK	MIC
Spearman's rho	pmrA	Correlation Coefficient	1	.759**	.571*	0.124	.405*	0.266	.471**
		Sig. (2-tailed)	.	0	0.021	0.484	0.017	0.128	0.005
	phoQ	Correlation Coefficient	.759**	1	.835**	-0.014	.435*	.510**	.766**
		Sig. (2-tailed)	0	.	0	0.937	0.01	0.002	0
	phoP	Correlation Coefficient	.571*	.835**	1	-0.269	.503*	0.487	.593*
		Sig. (2-tailed)	0.021	0	.	0.314	0.047	0.056	0.015
	mgrB	Correlation Coefficient	0.124	-0.014	-0.269	1	.382*	-0.121	-0.129
		Sig. (2-tailed)	0.484	0.937	0.314	.	0.026	0.495	0.468
	pmrC	Correlation Coefficient	.405*	.435*	.503*	.382*	1	.475**	0.224
		Sig. (2-tailed)	0.017	0.01	0.047	0.026	.	0.005	0.203
	pmrK	Correlation Coefficient	0.266	.510**	0.487	-0.121	.475**	1	.404*
		Sig. (2-tailed)	0.128	0.002	0.056	0.495	0.005	.	0.018
	MIC	Correlation Coefficient	.471**	.766**	.593*	-0.129	0.224	.404*	1
		Sig. (2-tailed)	0.005	0	0.015	0.468	0.203	0.018	.

** Correlation is significant at the 0.01 level (2-tailed). * Correlation is significant at the 0.05 level (2-tailed).

**Figure 3.**
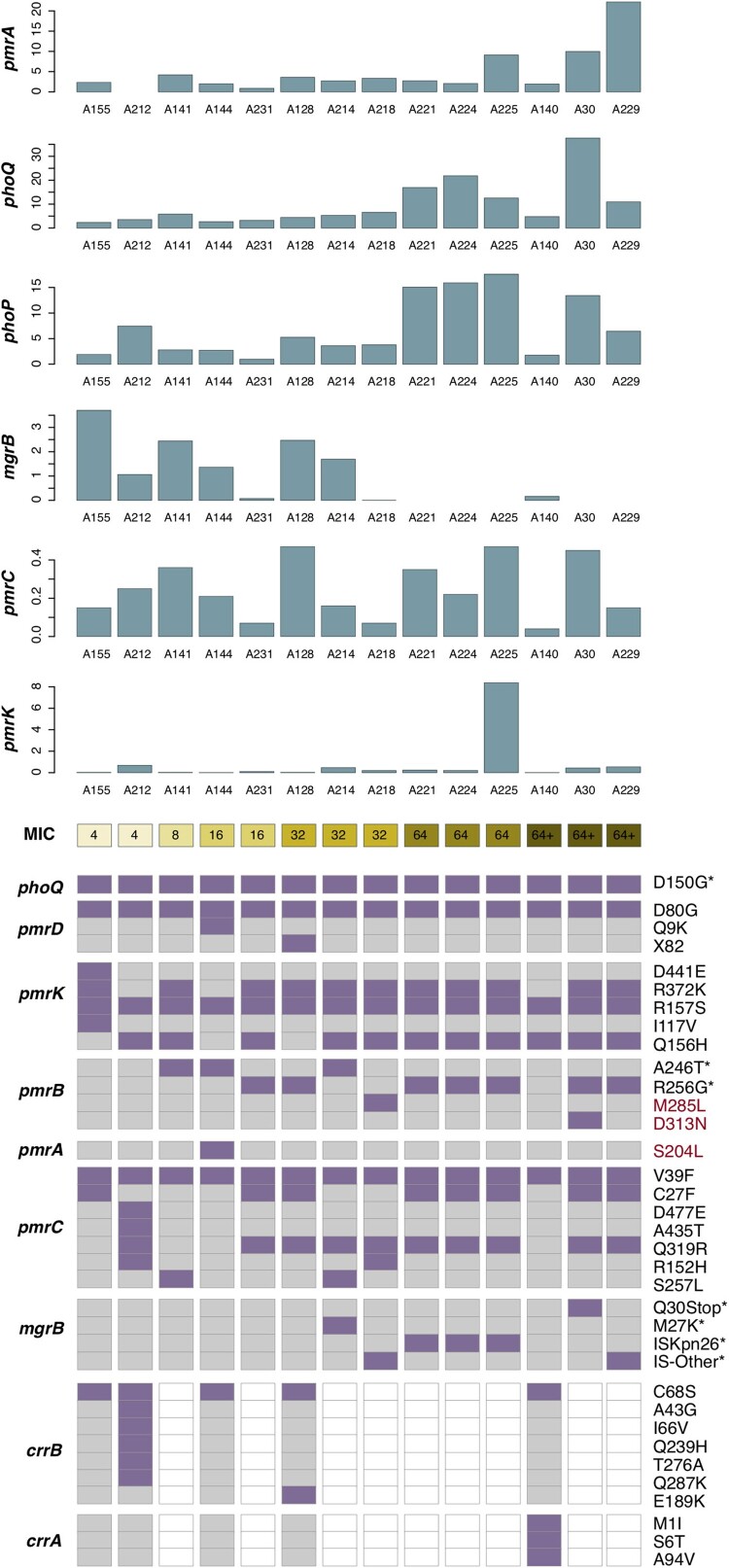
Transcriptional level and substitution mutations of chromosomal regulators in colistin-resistant isolates. The top six bar plots show the transcriptional level of six chromosomal regulators, with the *x*-axis represents 14 colistin-resistant isolates, ordered according to their colistin MICs, see the colour scale under the bar plot of *pmrK*. The prevalence of substitution mutations identified in nine chromosomal regulators are displayed in the heatmap following the colistin MICs. Each row shows a mutation site of corresponding gene (see the rightmost text), with mutated isolated colored in purple and those with no corresponding variation in grey. Genes absent in isolates are colored in white. Mutation reported in previous studies are marked with * in the rightmost. Three newly identified mutations which we speculated might have relation to colistin resistance are highlighted in red text.

Alteration in genome sequence is an important factor of transcription, which could determine the level of gene expression. Thus, we analyzed mutations of the genes responsible for colistin resistance. Compared with the reference genome of NTUH-K2044, 14 CoRKp strains presented no variation in *phoP* gene (named wild-type (WT)), while they all harboured D150G amino substitution in *phoQ* gene. In addition, six strains carried modified-*mgrB* containing Q30stop mutation and insert mutations mediated by IS*Kpn26* and other IS elements was detected (see Table 3 and Figure 3). M27 K mutation in *mgrB* has been reported to confer colistin resistance, which did not affect the expression of *mgrB* in our study. Additional mutations were identified in core genes of *pmrABCDK* and accessory genes *crrAB*.

**Table 3.** Colistin-resistant genes mediated by plasmid and mutations in the chromosomal genes in *K. pneumoniae.*

Name	COL mg/L	mcr	Amino acid alterations									
			phoQ	phoP	pmrD	pmrK	pmrB	pmrA	pmrC	mgrB	CrrB	CrrA
A155	4	-	D150G	WT	D80G	D441E R372K R157S I117V	WT	WT	V39F C27F	WT	**C68S**	+
A212	4	mcr-1.1	D150G	WT	D80G	R157S Q156H	WT	WT	D477E A435T Q319R R152H V39F	WT	**A43G I66V C68S Q239H T276A Q287K**	+
A141	8	mcr-1.1	D150G	WT	D80G	R372K R157S Q156H	**A246T**	WT	S257L V39F	WT		
A144	16	mcr-8.2	D150G	WT	D80G Q9K	R157S	**A246T**	S204L	V39F	WT	**C68S**	+
A231	16	-	D150G	WT	D80G	R372K R157S Q156H	R256G	WT	Q319R V39F C27F	WT		
A128	32	-	D150G	WT	X82 D80G	R372K R157S	R256G	WT	Q319R V39F C27F	WT	**C68S E189K**	+
A214	32	-	D150G	WT	D80G	R372K R157S Q156H	**A246T**	WT	Q319R S257L V39F	M27K		
A218	32	-	D150G	WT	D80G	R372K R157S Q156H	**M285L**	WT	Q319R R152H V39F	+		
A221	64	-	D150G	WT	D80G	R372K R157S Q156H	R256G	WT	Q319R V39F C27F	ISKpn26		
A224	64	-	D150G	WT	D80G	R372K R157S Q156H	R256G	WT	Q319R V39F C27F	ISKpn26		
A225	64	-	D150G	WT	D80G	R372K R157S Q156H	R256G	WT	Q319R V39F C27F	ISKpn26		
A140	>64	-	D150G	WT	D80G	R157S Q156H	WT	WT	V39F	WT	**C68S**	**M1I S6T A94V**
A30	>64	-	D150G	WT	D80G	R372K R157S Q156H	**D313N** R256G	WT	Q319R V39F C27F	Q30Stop		
A229	>64	-	D150G	WT	D80G	R372K R157S Q156H	R256G	WT	Q319R V39F C27F	+		

Abbreviations: COL: colistin; WT: wild-type.

Annotations of Amino acid alterations were based on the protein sequence of *Klebsiella pneumoniae subsp. pneumoniae* NTUH-K2044 (Accession no. NC_012731.1), except that CrrA, CrrB were based on the protein sequence of *Klebsiella pneumoniae subsp. pneumoniae* MGH 78578 (Accession no. NC_009648.1). The bold mutations were this article first found and reported, and the red mutation was that functional confirmation showed D313N could elevate the MIC of colistin.

### Functional confirmation of pmrA and pmrB novel point mutation

Except for two known mutations, R256G and A246 T in *pmrB* gene, we suspected that three novel variations, containing M285L and D313N in *pmrB* and S204L in *pmrA,* might contribute to colistin resistance (see Table 3). PCR and sequencing confirmed site-specific mutagenesis of amino acid substitution in *pmrAB*. The amino substitution D313N in *pmrB* in SZS128 elevated the MIC to 8 mg/L which was 16-fold higher than the original strain. However, M285L in *pmrB* and S204L in *pmrA* did not increase MICs of colistin against the original strain (see Sup Table 3).

### Identification of hvKp among colistin-resistant clinical strains

**Figure 4.**
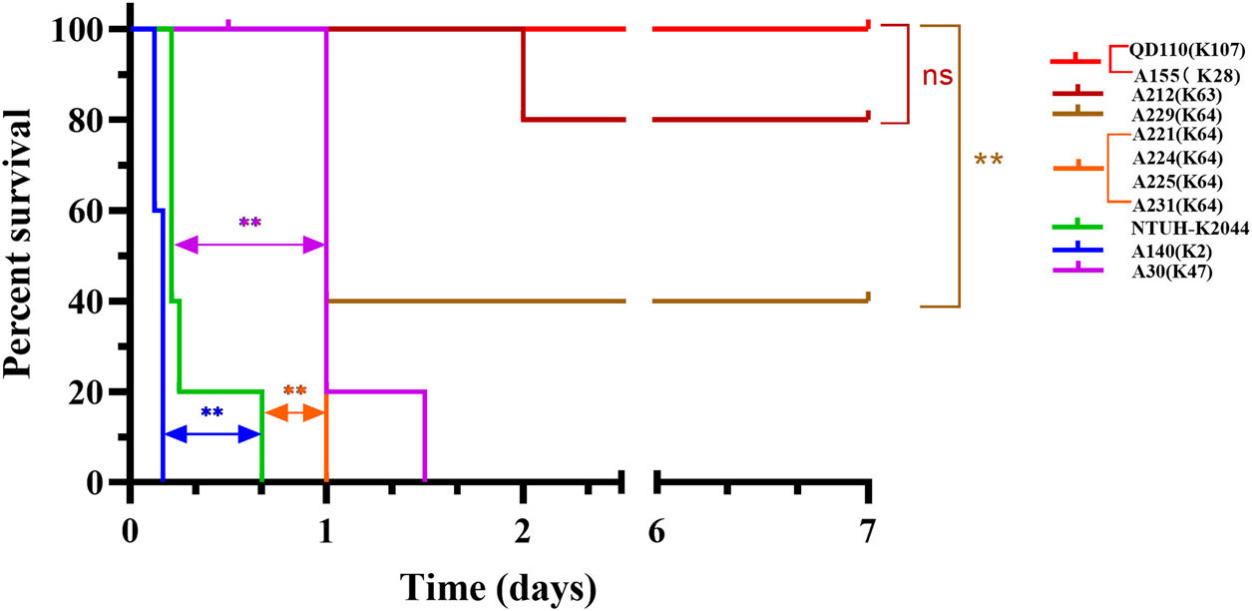
Survival curve of six weeks CD1 mice after IP infection with colistin-resistance *K. pneumoniae*. Each group, with five mice included with one *K. pneumoniae* strain, either 5×10^7^ CFU of 14 CoRKp strains or NTUH-K2044, or 1×10^8^ CFU of low-virulent control QD110. Strain of ST86-K2 presented the highest level of virulence, more virulent than the reference strain NTUH-K2044, and six strains from ST11-K64/K47 showed intermediate virulence, except for ST111-K63 isolate, which showed weak virulenceto some extent. Isolate from ST5253-K28 was classic virulence. Data of classic-virulent strains from, with no death of mice occurring within 7 days, were not shown, as the survival consistent with QD110. *P*-values from the log-rank (Mantel-Cox) test were indicated as follows: *, *P* < 0.05; **, *P* <0.01; ns, not significantly different.

Whole-genome sequencing was used to reveal the hypervirulent plasmid in CoRKp. The pLVPK-like virulence plasmid identified in 6 CoRKp strains harbouring the hypervirulent biomarker genes (*peg344*, *iroB*, *iucA, rmpA,* and *rmpA2)* were from ST11, ST86, and ST5253 strains (see Table 1 and Figure 1). The strain, A140, from ST86-K2 harbouring all five hypervirulent biomarkers showed the highest virulence potential. Three strains from ST11-K64 and one from ST11-K47 carrying hypervirulent genes showed middle-virulence between NTUH-K2044 and low-virulent control QD110 (see Figure 4). The strains, A221, and A225, carried *iroB, iucA, rmpA,* and *rmpA2* while A224 only carried *iroB* and *iucA* and A30 from K47 only carried *iucA* (see Table 1 and Figure 4). The hvKp from ST5253-K28 possessing *iucA* and *iroB* showed low virulence in mice IP model. Compared with related gene sequences in representative hvKp plasmid, the alignment identity of ST5253-K28 was among 78%−93%, which suggested a horizontal gene transcription event from other species (see Figure 1 and Sup Figure 2). A231 and A229 from ST11 strains lacked the hvKp marker genes mentioned above (see Sup Figure 3), while the virulent phenotype of these two strains was the same as strains from ST11 harboured hypervirulent genes (see Sup Figure 3 and Figure 4). Notably, *ybt* gene which encodes yersiniabactin was present in all six MDR strains in ST11 lineage. The strain from ST111-K63 harbouring *mcr-1.1* could lead to mice’s death in IP model in 24 h. The rest five isolates showed low virulence in mice IP model. Other virulence factors identified in the 14 colistin-resistant isolates were shown in Sup Figure 3. A significant positive correlation could be found in CoRKp (*P* < 0.0001) by using Mantel–Haenszel *χ*^2^-test to reveal the association between virulence and MICs.

### Biofilm-forming capacity in colistin-resistant strains

The mean OD590 nm values obtained by the quantitative biofilm-production assay were itemized in Table 1. Four strains were strong biofilm producers. Five strains showed moderate biofilm-forming capacities. Two strains were weak biofilm producers and the rest two strains showed non-biofilm capabilities. We regarded strains with colistin MIC ≥8 mg/L as high MIC group, while others with MIC <8 mg/L as low MIC group. The results showed that high MIC group of CoRKp showed lower biofilm-forming capacity (0.6647 ± 0.12494) than low MIC group (1.1673 ± 0.86826).

### Transcriptome analysis of strong biofilm producers of CoRKp

**Figure 5.**
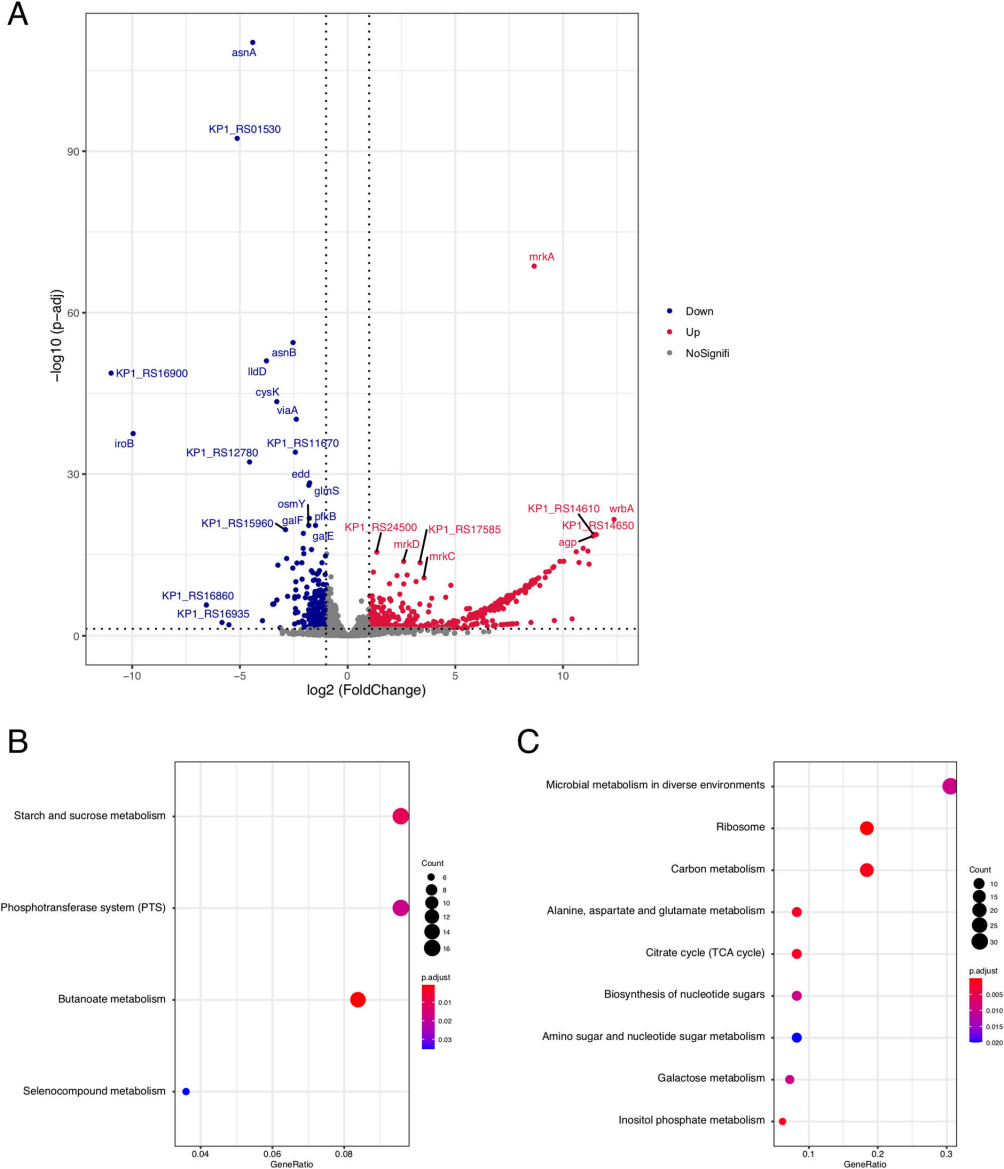
Differently expressed genes between colistin-resistant and colistin-sensitive isolates. (A) Volcano plots of the differently expressed genes. (B) Point plot of KEGG pathways for up-regulated genes. (C) Point plot of KEGG pathways for down-regulated genes.

In order to investigate DEGs of strong biofilm producers isolates, we selected three isolates with strong biofilm-forming ability and a weak biofilm producer strain, ATCC13883, for RNA-seq analysis. A total of 623 DEGs (with absolute log2 fold change >1 and p. adjust < 0.05) were identified, including 416 up-regulated genes and 207 down-regulated genes (Figure 5A and Supplementary Table 1). The most significant up-regulated gene *mrkA* whose log_2_^fold change^ was 8.64, encodes type III fimbrial shaft MrkA, which could facilitate biofilm formation [37]. Meanwhile, for down-regulated genes, *asnA* encoding aspartate-ammonia ligasem and *KP1_RS01530* encoding lytic transglycosylase F, was differentially expressed. The up-regulation of *asnA* was reported to be involved in biofilm formation of *Streptococcus pneumoniae* [39]. For biological functions of these differently expressed genes, 416 up-regulated genes were enriched in 4 KEGG pathways, i.e. starch and sucrose metabolism, phosphotransferase system (PTS), butanoate metabolism and selenocompound metabolism (Figure 5B), while the 207 down-regulated genes were enriched in 9 KEGG pathways, including the most enriched pathways of microbial metabolism in diverse environments, ribosome, and carbon metabolism (Figure 5C).

## Discussion

Colistin could be used to cure the infection caused by MDR *K. pneumoniae*, but the misuse of colistin, in turn, has caused a prevalence of colistin resistance (CHINET data, 2017–2020. http://www.chinets.com/Data/AntibioticDrugFast). The 14 colistin-resistant isolates were assigned to various lineage, which indicates that the CoRKp has a wide genetic diversity and geographic distribution. ST11 was the dominant sequence type of CoRKp. The MDR percentage of CoRKp strains in our study was 92.8%, which will bring difficulties to further clinical treatment. One PDR strain was determined from ST11 isolated from blood, which highlights the increasing multidrug resistance in clinical *K. pneumoniae* isolates in China. These findings suggested that colistin-resistant *K. pneumoniae* has become a serious public problem.

In addition, only three of the 14 colistin-resistant isolates carried mobile colistin-resistance gene, *mcr-1.1* or *mcr-8.2*, with different colistin MICs. The *mcr* prevalence in colistin-resistant *K. pneumoniae* (21%) was significantly lower than that in *E. coli* (87.5%, data unpublished). Unlike the first reported *mcr-8.2*-bearing strain in ST395 isolated from animal [40], the first *mcr-8.2*-positive clinical isolate in our study was ST2459, a novel *K. pneumoniae* ST submitted by our study.

Compared with plasmid-mediated colistin resistance by *mcr*, the overexpression of TCSs regulator genes played a more important role in resistance. Our results speculated that *phoPQ* regulatory factors contributed most to chromosome-mediated colistin resistance in *K. pneumoniae* because of correlation. Previous reports revealed that the various overexpression of TCSs such as *pmrAB* [41], *phoPQ* [41], *pmrC* [42], and *pmrK* [42,43] might lead to colistin resistance, but it is still unclear which regulator is the most important one. Besides, multiple linear regression equation was first proposed in our study, which could intuitively reflect that *pmrA* and *phoQ* played important roles in colistin resistance. Whereas, the formula still needs clinical isolates with various MICs to verify. In conclusion, the chromosome-mediated resistance to colistin in *K. pneumoniae* was mainly caused by up-regulated expression *phoPQ* system, which was proved to be a key element affecting MICs.

Several mutations in chromosomal regulators were detected by the whole-genome analysis . Modified-*mgrB*, including insertion of diverse IS fragments and amino acid substitution are now primary colistin-resistance mechanisms in *K. pneumoniae* [44–46]. In addition, amino acid substitutions, such as T157P in *pmrB* [47], D191Y in *phoP* [48] and L26P in *phoQ* [49] were reported to increase MICs by 24–1000 folds among clinical isolates, while D150G in *phoQ,* a common mutation in CoRKp, could not impact colistin resistance [49]. In this study, 3 unreported substitutions were found. As these mutations had correlation of expression of *pmrA*, we speculated that they might contribute to colistin resistance. D313N in *pmrB* was verified as an available mutation in colistin resistance. The rest of unreported mutations in *pmrA* and *pmrB* did not contribute to resistance. D313N and M285L were located on catalytic and ATP-binding (CA) domain in *pmrB*. M285L was predicted as a neutral mutation by PROVEAN (http://provean.jcvi.org/index.php). D313N was predicted as a deleterious mutation that was speculated to enhance the ATP-binding capability [50], thus could increase the MIC of colistin. A recent study reported that a missense mutation in *crrB* contributed to colistin resistance [51]. However, it was an accessory genome and was not detected in all colistin-resistant strains [44]. Thus, the expression of *crrAB* system was not taken into consideration in our study. According to our results, we speculated that colistin resistance in *K. pneumoniae* results from a combination of multiple mutations, although the molecular mechanisms of some amino substitutions are still unknown. Further investigations are needed to confirm the relationship between these mutations and colistin resistance.

Consistent with previous researches, we found a cluster of colistin- and carbapenem-resistant *K. pneumoniae* (CoR-CRKp) strains in ST11 lineage and most of them were hvKp as well [52]. Premature stop mutation and insertion sequence (mainly IS*Kpn26*) in *mgrB* were the main cause of colistin resistance. Importantly, previous study characterized IS*Kpn72* element in a mobile plasmid and chromosomal gene, which suggested chromosomal colistin-resistant mechanism could be transmitted by plasmid [53]. In further investigations, we should focus on whether all types of insertion sequences could lead to the transmission of IS elements to chromosomes. Besides, it is reported that *phoPQ* system and *mgrB* could elevate virulence. *mgrB* inactivation of *K. pneumoniae* increases virulence in *Galleria mellonella* infection model [54], and deletion of *phoPQ* could decrease invasion ability in *Shigella flexner* [55]. We used hypervirulent biomarkers to identify the hvKp, and 83.3% hv-CoRKp showed virulence in mice IP model. Instead, *ybt* seems not to play a particular role because of the existence in all ST11 isolates. Brazil reported a ST11-K64 *K. pneumoniae* fatal bacteremia while isolates without hypervirulent biomarkers [56], as our two ST11-K64 CoRKp (A229 and A231) also showed virulent phenotype speculated that capsule might also correlate with virulence. Furthermore, we found that MICs had a positive correlation with virulence, consistent with the report in CoRKp of various sequence types [57]. The emergence of modified-*mgrB-*mediated colistin-resistant *K. pneumoniae* should be monitored to prevent it from becoming a severe public health challenge.

We also studied the biofilm-forming capacity of colistin against *K. pneumoniae in vitro* and found that it was reduced in isolates with high colistin MICs, which was consistent with several reports in other pathogens. As it was documented, the biofilm formation was significantly reduced in colistin-resistant isolate in *Salmonella typhimurium,* when compared with colistin-susceptible isolates [58]. The conclusion in *Acinetobacter baumannii* was the same*.* Both *in vitro* and *vivo* analysis revealed that isolates with lower MICs had stronger biofilms which were associated with mutations in colistin-modified TCS *pmrB* [59]. Besides, we speculated there were some relationships between biofilm and TCSs. In *Pseudomonas aeruginosa*, a study showed that multiple extracellular DNA acidify in biofilm could induce the expression of *phoPQ* and *pmrAB*. However, it did not reveal whether colistin resistance could elevate biofilm formation capability [60]. We conjectured that there would be a similar phenotype in *K. pneumoniae*, and more researches are still needed. In this study, we used RNA-seq to reveal genes differentially express between strong biofilm producers in CoRKp and ATCC13883. We found *mrkA*, *mrkD* and *mrkC* were significantly up-regulated, which all participated in type III fimbria [61]. Our study showed that colistin-resistant MDR strains could have strong biofilm-forming ability which might make colonized *K. pneumoniae* more difficult to treat.

In conclusion, we found a low prevalence of *mcr* genes in CoRKp during 2017–2018 in China, which means that *chromosome*-mediated mechanism was still the main cause of colistin-resistance in *K. pneumoniae.* The up-regulated expression of TCS, especially *phoPQ,* played an essential role in colistin-resistant mechanism. And *mgrB* inactivation was a major contributor to the overexpression of chromosomal regulators. Additionally, most of the CoR-CRKp strains, clustered in ST11 lineage, were hvKp as well, which might be associated with the currently reported high mortality rate in CoR-CRKp. Thus, continuous surveillance of *K. pneumoniae*, especially hypervirulent CoR-CRKp, is an urgent priority to help prevent their spread.

## Supplementary Material

Supplemental Material

## Data Availability

The raw data of both whole-genome DNA and RNA sequencing data have been deposited in the NCBI Sequence Read Archive under BioProject ID PRJNA762527.
