# Supplementary material for: Emergence of colistin-resistant hypervirulent Klebsiella pneumoniae (CoR-HvKp) in China
Source: Emerg Microbes Infect. 2022 Mar 3;11(1):648–61. doi: 10.1080/22221751.2022.2036078 (PMC8896207; doi:10.1080/22221751.2022.2036078)
Supplement: Supplemental Material [file TEMI_A_2036078_SM4192.zip › Suppl files/Sup Fig_clean.docx]

Supplementary Information for **“Emergence of colistin-resistant hypervirulent Klebsiella pneumoniae (CoR-HvKp) in China”**

**Table S2.** Primers used in the functional verification of *pmrA* and *pmrB* point mutation.

| Function | Name | Sequence (5' to 3') | description |
| --- | --- | --- | --- |

| *pmrA* gene G611T mutation in *K. pneumoniae* SZS128 strain | pmrA-1_spacerF | TAGTCACAACCTACGCGAAAAGAT | *pmrA* spacer for gene partial deletion |
| --- | --- | --- | --- |
|  | pmrA-1_spacerR | AAACATCTTTTCGCGTAGGTTGTG |  |
|  | dspmrA1-re1 | AGAAACATGTGCCGATGGCG | amplification of ~ 500 bp *pmrA* upstream for gene partial deletion |
|  | dspmrA1-2 | AGAAGACGCTAGTTCCTCCAGGGCAAAGG |  |
|  | dspmrA1-3 | TGGAGGAACTAGCGTCTTCTGGCTATGGCA | amplification of ~ 500 bp *pmrA* downstream for gene partial deletion |
|  | dspmrA1-re4 | TGCTGGTGGTCATCTGATCG |  |
|  | pmrA-2_spacerF | TAGTCTGGAGGAACTAGCGTCTTC | *pmrA* spacer for sequence complementation |
|  | pmrA-2_spacerR | AAACGAAGACGCTAGTTCCTCCAG |  |
|  | dspmrA2-2 | TGCCATAGCCAGAAGACGCT | amplification of 987 bp *pmrA* upstream for gene complementation accompanied with primer: dspmrA1-re1 in *K. pneumoniae* A144 strain |
|  | dspmrA2-3 | AGCGTCTTCTGGCTATGGCA | amplification of 484 bp *pmrA* downstream for gene complementation accompanied with primer: dspmrA1-re4 in *K. pneumoniae* SZS128 |
|  | dspmrA1-1 | GTGAAGGATACGTGTGCGAC | sequencing of *pmrA* mutation |

| *pmrB* gene A853T mutation in *K. pneumoniae* SZS128 strain | pmrB-1_spacerF | TAGTCAGTAAAGCCTTCGTGCGCA | *pmrB* spacer for gene partial deletion |
| --- | --- | --- | --- |
|  | pmrB-1_spacerR | AAACTGCGCACGAAGGCTTTACTG |  |
|  | dspmrB1-re1 | AAAGCGAACTGGAGAACCGC | amplification of ~ 500 bp *pmrB* upstream for gene partial deletion |
|  | dspmrB1-2 | TGGTCATTATAGATTGCGCAGGATCACGC |  |
|  | dspmrB1-3 | TGCGCAATCTATAATGACCAGGGCGGTGG | amplification of ~ 500 bp *pmrB* downstream for gene partial deletion |
|  | dspmrB1-re4 | GTGCCGCAGGGGCTCAATAA |  |
|  | pmrB-2_spacerF | TAGTCTGGTCATTATAGATTGCGC | *pmrB* spacer for gene complementation |
|  | pmrB-2_spacerR | AAACGCGCAATCTATAATGACCAG |  |
|  | dspmrB2-2 | AGATTGCGCAGGATCACGC | amplification of 487 bp *pmrB* (A853T) upstream for gene complementation accompanied with primer: dspmrB1-re1 in *K. pneumoniae* SZS128 strain |
|  | dspmrB2-3 | GCGTGATCCTGCGCAATCT | amplification of 850 bp *pmrB* (A853T) upstream for gene complementation accompanied with primer: dspmrB1-re4 in *K. pneumoniae* A218 strain |
| *pmrB* gene G937A mutation in *K. pneumoniae* SZS128 strain | pmrB-1_spacerF | TAGTCAGTAAAGCCTTCGTGCGCA | *pmrB* spacer for gene partial deletion |
|  | pmrB-1_spacerR | AAACTGCGCACGAAGGCTTTACTG |  |
|  | dsB-F | GCGTGATCCTGCGCAATCT | amplification of 220 bp *pmrB* (containg G937A) in *K. pneumoniae* A30 strain |
|  | dsB2-R | ATGCGCGTGACGATGCTCA |  |
|  | dspmrB1-1 | CTGCTGGCGAAAGTGCATGG | sequencing of *pmrB* mutation |

**Table S3.** MICs colistin against of SZS128, SZS128-*pmrA*^S204L^, SZS128-*pmrB*^M285L^ and SZS128-*pmrA*^D313N^

| Strain | mutation in *pmrAB* | Colistin MIC (mg/L) |
| --- | --- | --- |

| SZS128 | WT | 0.5 |
| --- | --- | --- |
| SZS128-*pmrA^S204L^* | *pmrA^S204L^* | 0.5 |
| SZS128-*pmrB^M285L^* | *pmrB^M285L^* | 0.5 |
| **SZS128-*pmrB^D313N^*** | ***pmrB^D313N^*** | **8** |

**
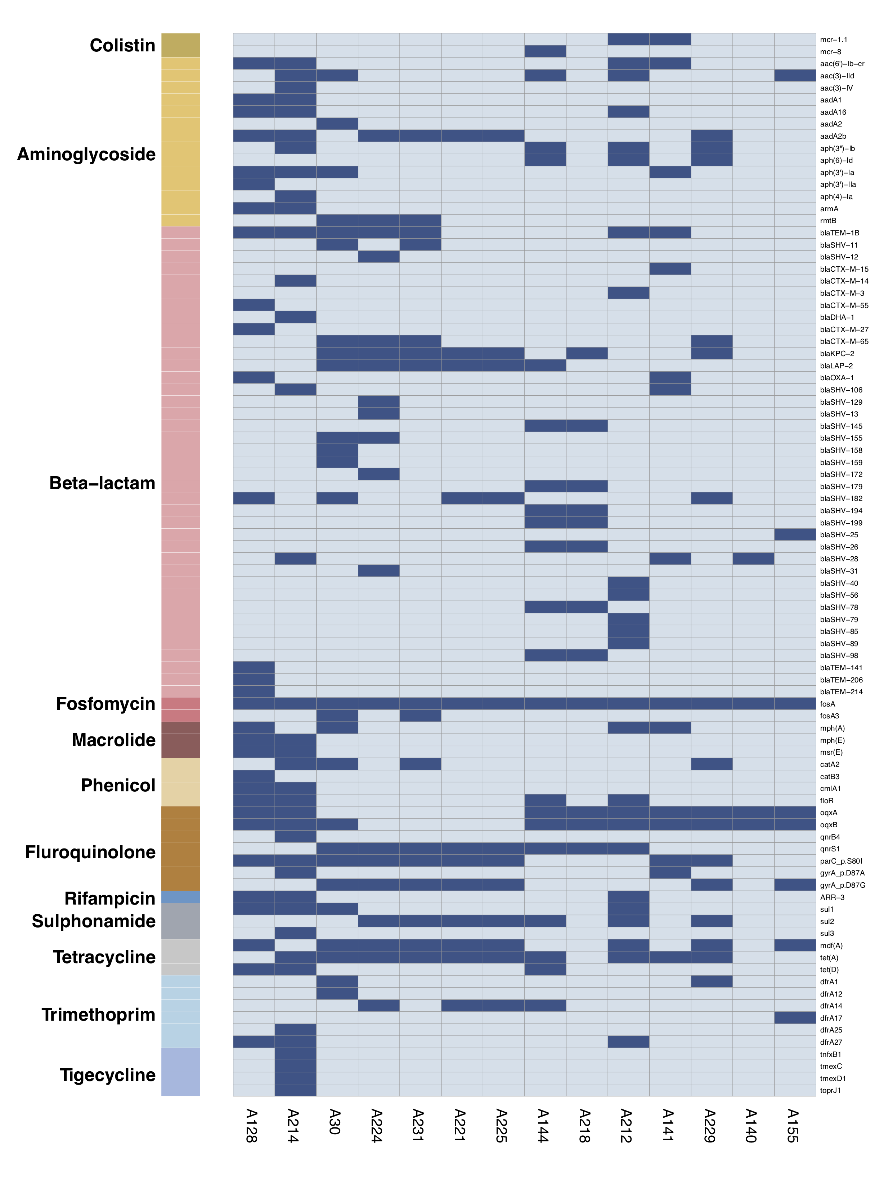
**

**Sup Fig 1.** Heatmaps of antibiotic resistance genes identified in 14 colistin-resistant isolates. The bar on the left represents common resistance mechanisms, and the text on the rightmost shows specific gene names. The heatmap displays the presence and absence of antibiotic resistance genes or mutations in each isolate, aligned with the public database of ResFinderand PointFinder. Each column of the heatmap represents a colistin-resistant isolate. Genes present in the isolate are colored in dark blue, while genes absent colored in light blue.

**
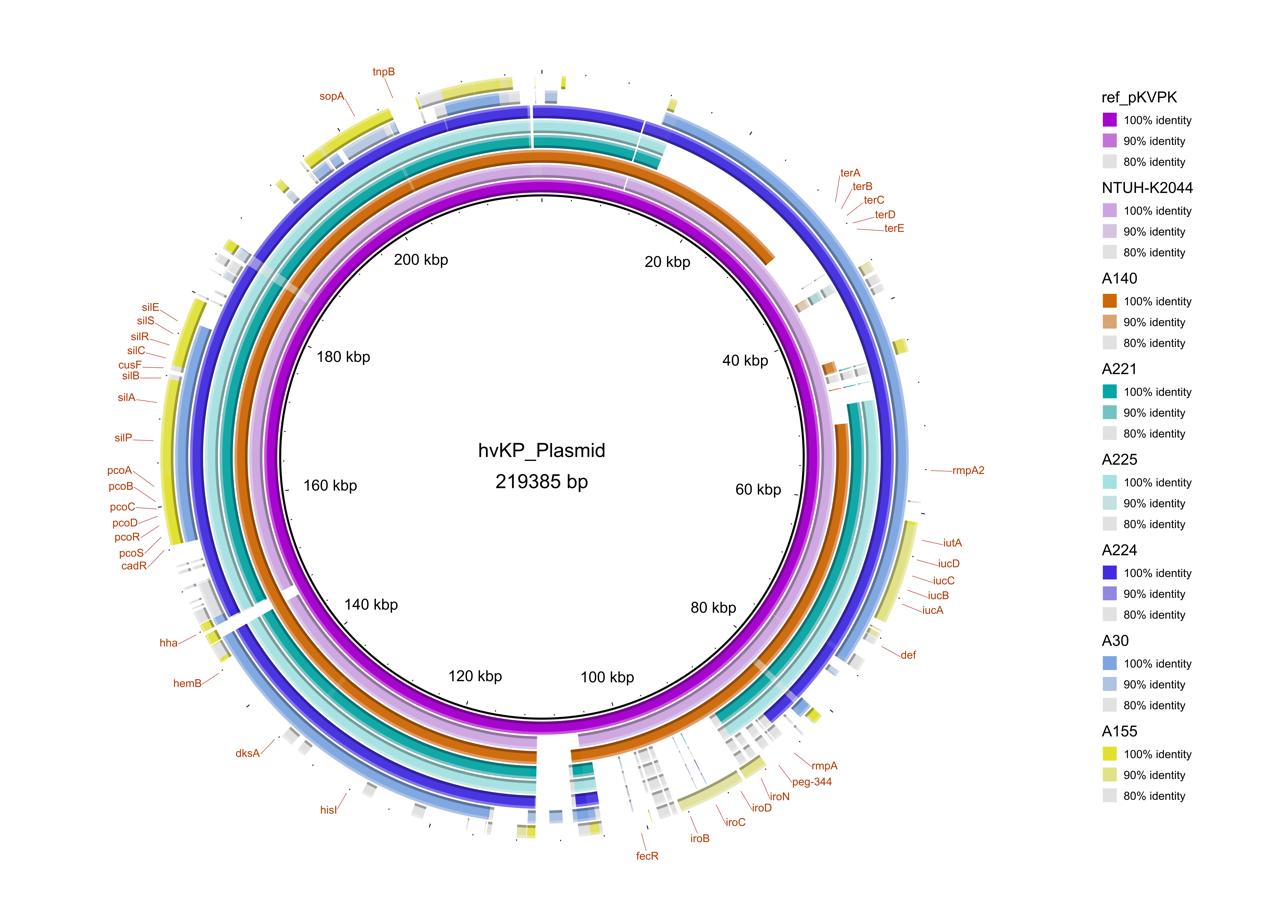
**

**Sup Fig 2.** Genome comparations of hvKp plasmids. The innermost ring represents the reference plasmid of pLVPK. And the other seven rings represent NTUH-K2044 and six newly identified hvKp isolates, respectively, with color scale represents the identity of BLAST comparations (see the rightmost legend). White space in each ring represents the fragments existed in the reference are lost in this isolate. Annotated gene name in genome annotation file of pLVPK are labeled in red text in the outmost.

**
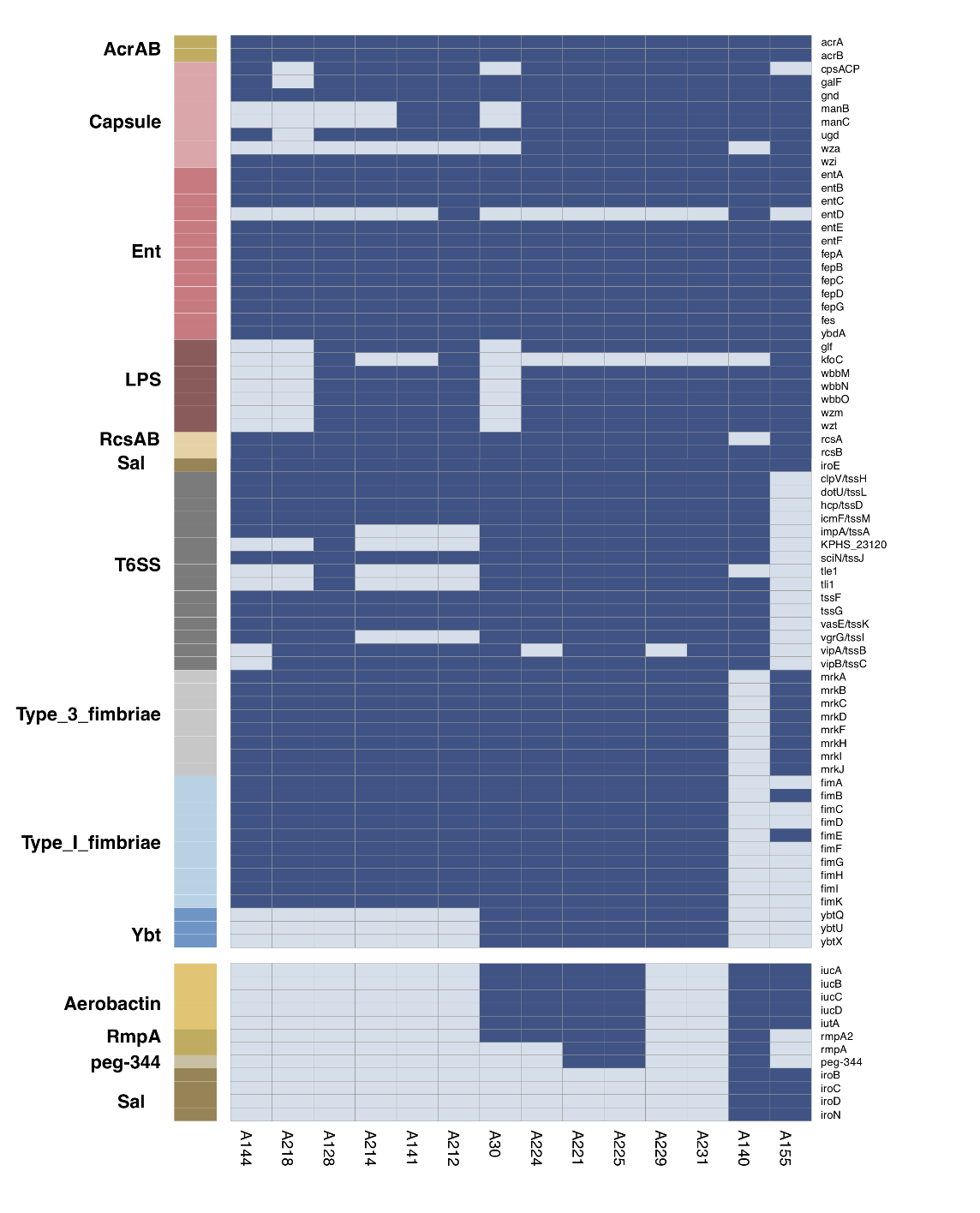
**

**Sup Fig 3.** Heatmaps of virulence factors identified in 14 colistin-resistant isolates. The bar on the left represents common classifications of virulence factors, and the text on the rightmost shows specific virulence gene names. The heatmap displays the presence and absence of virulence factors in each isolate, aligned with VFDB database. Each column of the heatmap represents a colistin-resistant isolate. Genes present in the isolate are colored in dark blue, while genes absent colored in light blue. In order to have a comprehensive display of identified virulence genes, hvKp-related genes, consistent with that in Figure 2, were shown again in the lower part of the heatmap, with whitespace separated with the upper part.

**
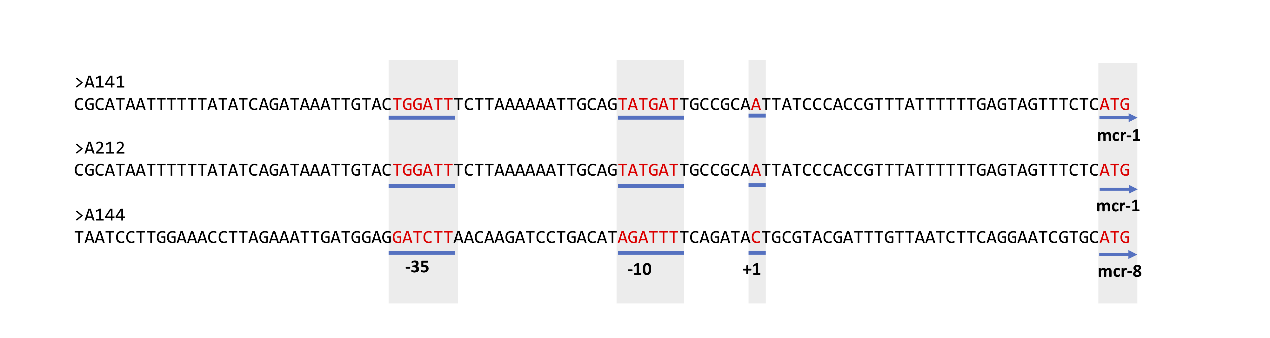
**

**Sup Fig 4.** Alignment of promoter sequences of the *mcr-1* and *mcr-8* genes from three clinical isolates. The -35, -10, +1 region and start codon of the promoter sequences are highlighted in grey boxes.

**Sup Fig 5.** Correlation analysis of colistin MICs and transcription levels of chromosomal regulators. (A) Relationship between colistin MICs and the expression of regulators. X-axis was log_2_MIC of each isolate and Y-axis was the expression level of regulators. (B) Relationship of expressions between different regulators, with solid line representing the fitted straight line, and dotted line indication confidence intervals. Transcription levels of *pmrA*, *phoQ*, *mgrB*, *pmrC*, *pmrK* and *phoP* were detected by RT-qPCR and were normalized to levels of *rpoB*.
