# Supplementary figures and images for: Emergence of colistin-resistant hypervirulent Klebsiella pneumoniae (CoR-HvKp) in China
Source: Emerg Microbes Infect. 2022 Mar 3;11(1):648–61. doi: 10.1080/22221751.2022.2036078 (PMC8896207; doi:10.1080/22221751.2022.2036078)

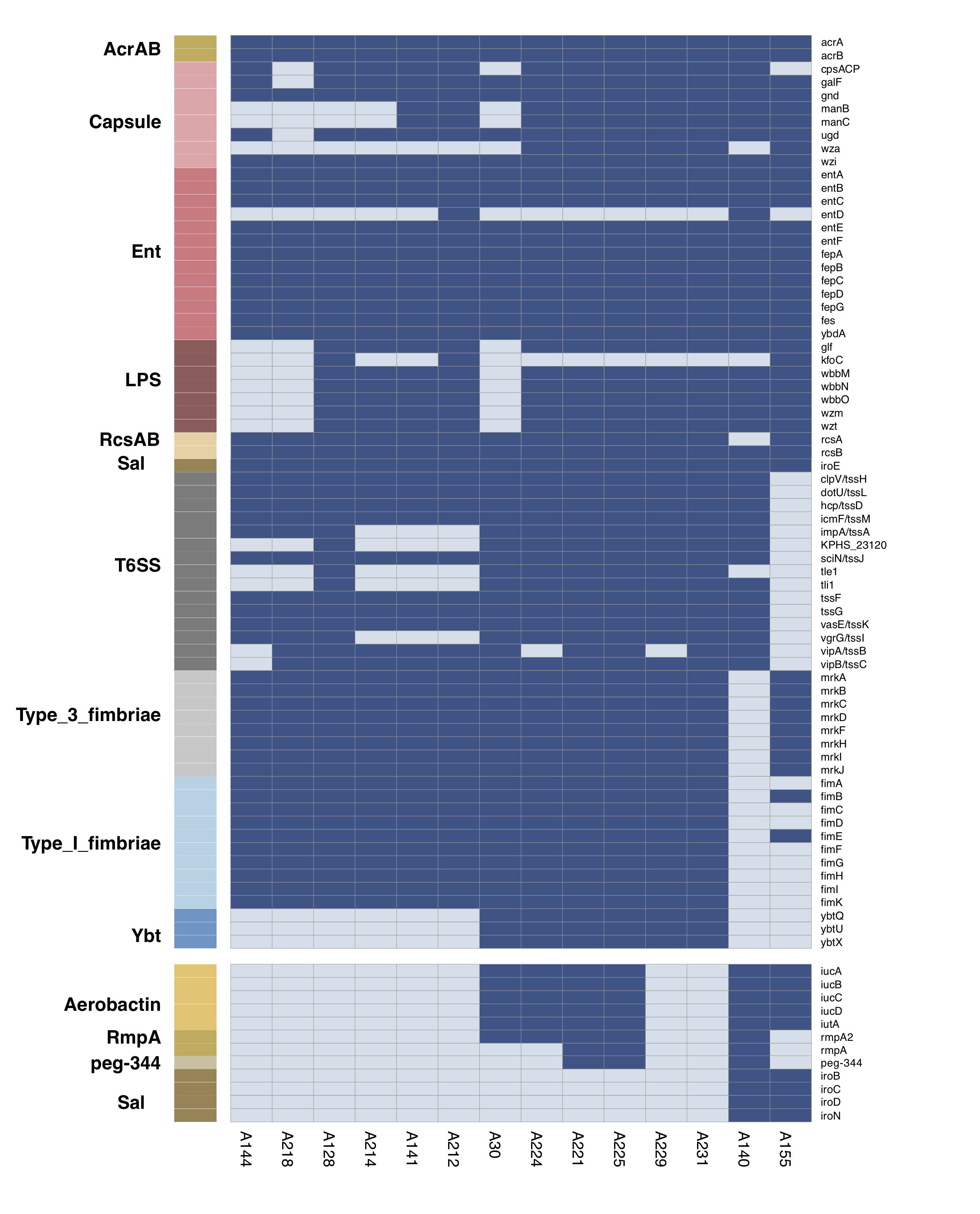

Supplement: Supplemental Material [file TEMI_A_2036078_SM4192.zip › Suppl files/sup fig 3.tiff]

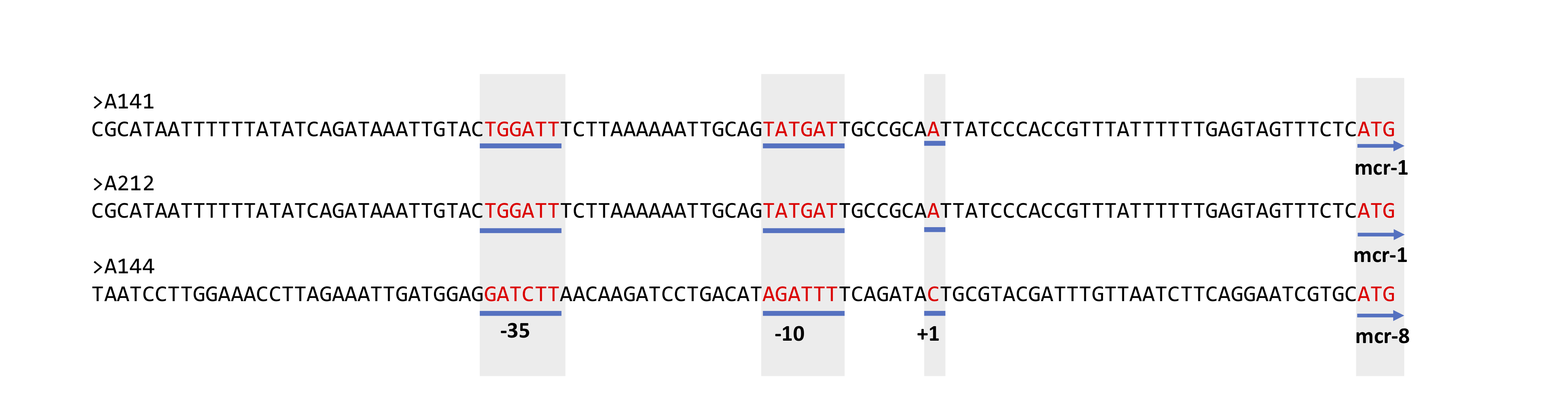

Supplement: Supplemental Material [file TEMI_A_2036078_SM4192.zip › Suppl files/sup fig 4.tiff]

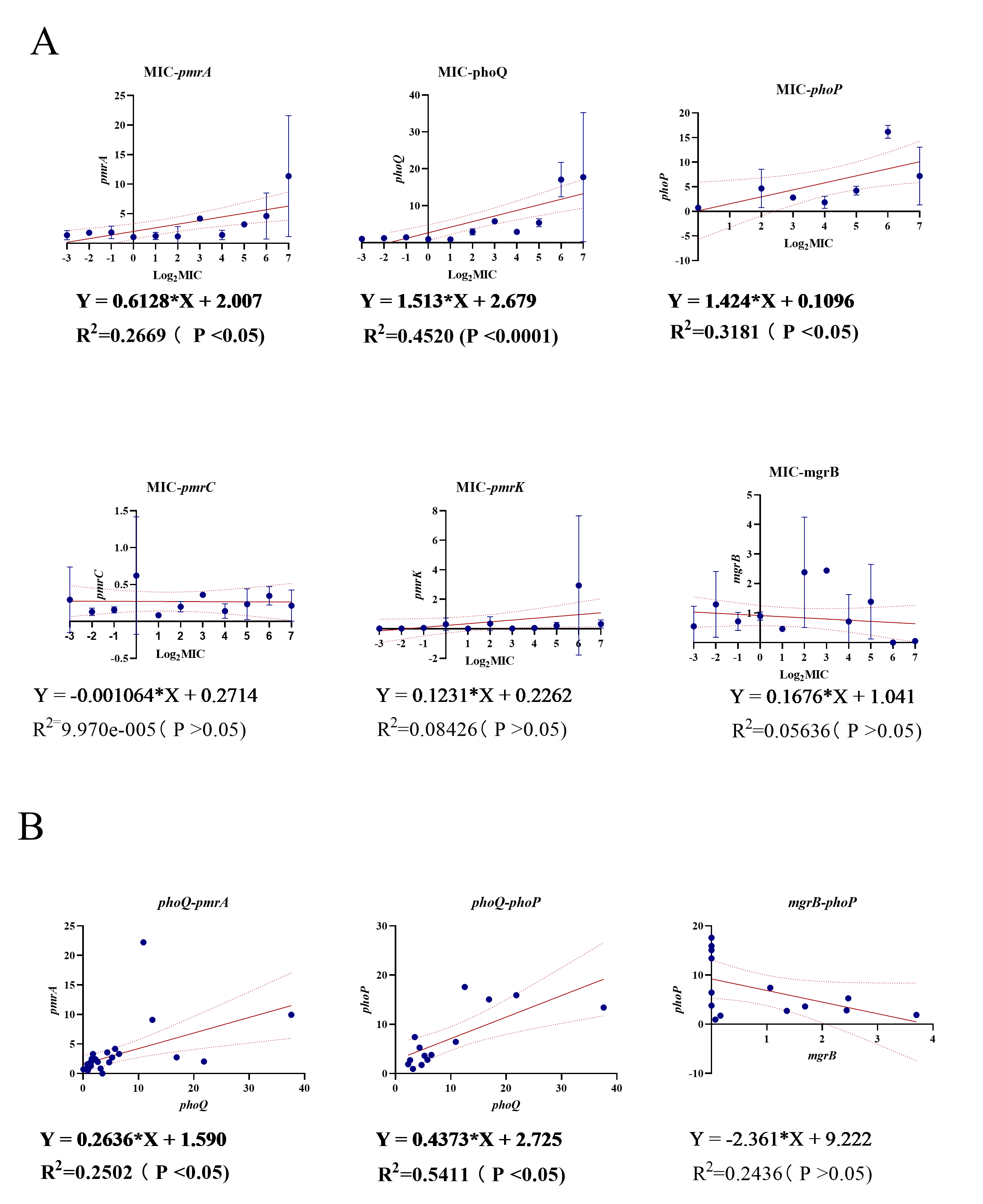

Supplement: Supplemental Material [file TEMI_A_2036078_SM4192.zip › Suppl files/sup fig 5.tif]

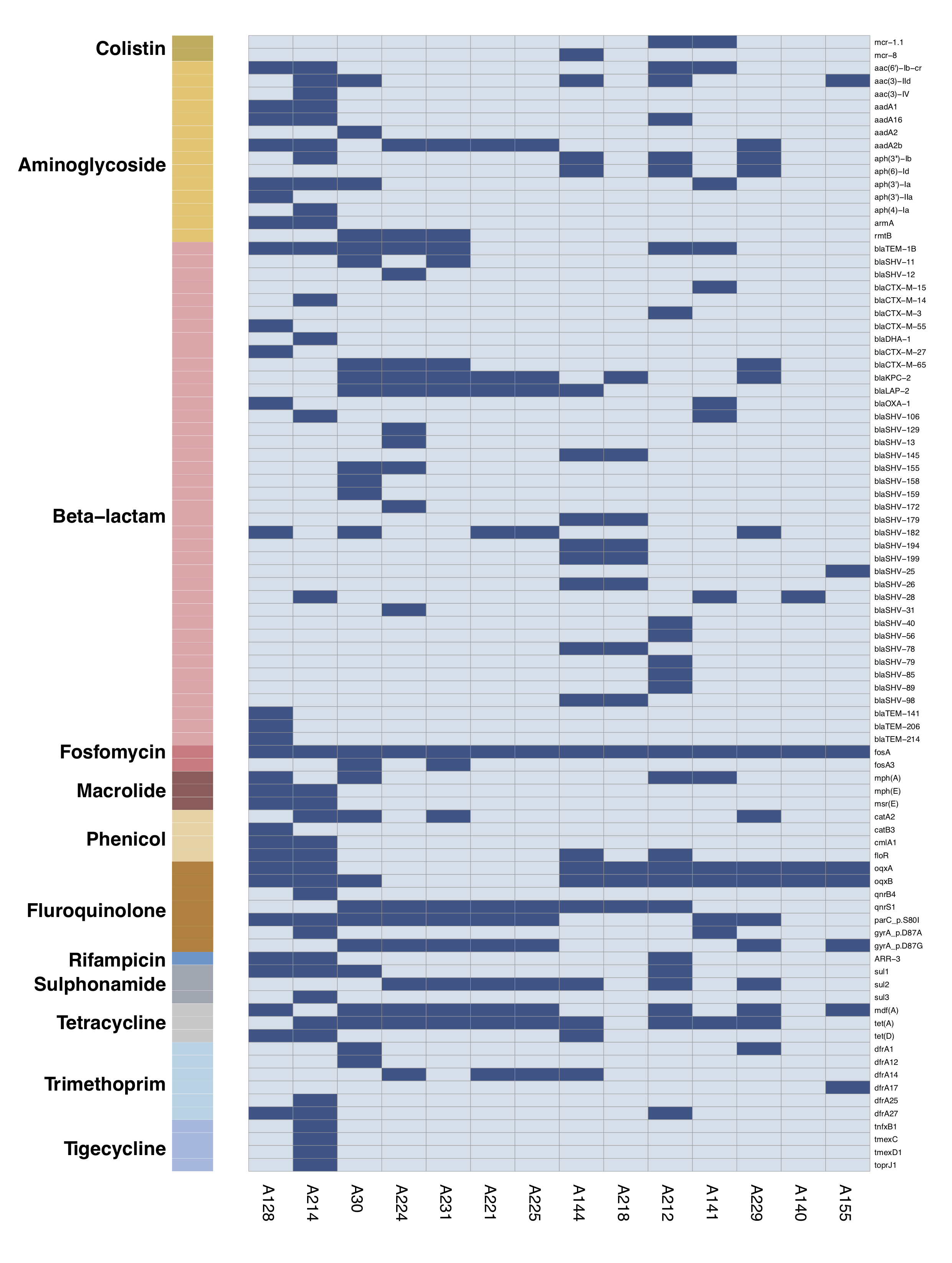

Supplement: Supplemental Material [file TEMI_A_2036078_SM4192.zip › Suppl files/sup fig1.tiff]
